# Evaluating the potential for multi-generational longitudinal research using U.S. Census data from 1850-2020

**DOI:** 10.23889/ijpds.v9i5.2843

**Published:** 2024-09-18

**Authors:** Katie Genadek, Trent Alexander

**Affiliations:** 1 U.S. Census Bureau; 2 University of Colorado; 3 University of Michigan

Multiple recent linkage efforts have produced longitudinal individual-level data from the censuses of 1850-1940. In addition, within the Census Bureau’s restricted data program, respondents can be linked anonymously between the censuses of 1940, 2000, 2010, and 2020. Researchers are already using parts of these infrastructures together to conduct long-term inter-generational studies. In this paper we describe the various options for accessing, linking, and conducting research that uses both the historical and modern linked data as a centuries-long panel. We assess the sample sizes, representativeness, and cohort coverage of multigenerational panels created with existing linked census resources over the 1850-2020 period.

